# Dietary Compound Kaempferol Inhibits Airway Thickening Induced by Allergic Reaction in a Bovine Serum Albumin-Induced Model of Asthma

**DOI:** 10.3390/ijms161226218

**Published:** 2015-12-16

**Authors:** Daekeun Shin, Sin-Hye Park, Yean-Jung Choi, Yun-Ho Kim, Lucia Dwi Antika, Nurina Umy Habibah, Min-Kyung Kang, Young-Hee Kang

**Affiliations:** Department of Food and Nutrition, Hallym University, Chuncheon 200-702, Korea; aceflavor@hotmail.com (D.S.); sinhyepark@hallym.ac.kr (S.-H.P.); yeanjungchoi@gmail.com (Y.-J.C.); royalskim@hallym.ac.kr (Y.-H.K.); lucia.dwiantika@gmail.com (L.D.A.); nurinahabibah@gmail.com (N.U.H.); mitholy@hallym.ac.kr (M.-K.K.)

**Keywords:** airway thickening, asthma, bronchoconstriction, cyclooxygenase 2, kaempferol

## Abstract

Asthma is characterized by aberrant airways including epithelial thickening, goblet cell hyperplasia, and smooth muscle hypertrophy within the airway wall. The current study examined whether kaempferol inhibited mast cell degranulation and prostaglandin (PG) release leading to the development of aberrant airways, using an *in vitro* model of dinitrophenylated bovine serum albumin (DNP-BSA)-sensitized rat basophilic leukemia (RBL-2H3) mast cells and an *in vivo* model of BSA-challenged asthmatic mice. Nontoxic kaempferol at 10–20 μM suppressed β-hexosaminidase release and cyclooxygenase 2 (COX2)-mediated production of prostaglandin D2 (PGD2) and prostaglandin F2α (PGF2α) in sensitized mast cells. Oral administration of ≤20 mg/kg kaempferol blocked bovine serum albumin (BSA) inhalation-induced epithelial cell excrescence and smooth muscle hypertrophy by attenuating the induction of COX2 and the formation of PGD2 and PGF2α, together with reducing the anti-α-smooth muscle actin (α-SMA) expression in mouse airways. Kaempferol deterred the antigen-induced mast cell activation of cytosolic phospholipase A2 (cPLA2) responsive to protein kinase Cμ (PKCμ) and extracellular signal-regulated kinase (ERK). Furthermore, the antigen-challenged activation of Syk-phospholipase Cγ (PLCγ) pathway was dampened in kaempferol-supplemented mast cells. These results demonstrated that kaempferol inhibited airway wall thickening through disturbing Syk-PLCγ signaling and PKCμ-ERK-cPLA2-COX2 signaling in antigen-exposed mast cells. Thus, kaempferol may be a potent anti-allergic compound targeting allergic asthma typical of airway hyperplasia and hypertrophy.

## 1. Introduction

Allergic responses, including itching, runny nose, coughing, and trouble breathing, commonly occur following the invasion of allergens [1,2,3]. Among the various allergic diseases, bronchial asthma causes an anaphylactic response triggered by the cross-linking of high-affinity Fc receptors for immunoglobulin E (IgE) located on the surface of mast cells and basophils [4,5]. It is found that upon the stimulation of both mast cells and basophils through the activation of IgE receptors chemicals, such as histamine and serotonin, are released to evoke acute airway inflammation [6,7]. Activation of mast cells by aggregation of their IgE receptors induces rapid and transient synthesis of cyclooxygenase 2 (COX2) [8,9]. In addition, mast cells are crucial effector cells in allergic disorders, releasing lipid mediators and eicosanoids, including prostaglandins (PGs) and leukotrienes, that cause airway constriction via smooth muscle contraction [10,11]. Prostaglandin D2 (PGD2) is a major PG produced by mast cells, recruits Th2 cells, eosinophils, and basophils and is critical to the development of allergic diseases [12]. Prostaglandin F2α (PGF2α) exerts the potent secretagogue activity of the mucus secretion in bronchi and trachea, stimulates the contraction of uterine and bronchial smooth muscle, and participates in the inflammation modulation and blood vessel vasoconstriction [13].

Cytosoli phospholipase A2 (cPLA2) catalyze the hydrolysis of the sn-2 position of cell membrane glycerophospholipids, leading to production of free fatty acids and lysophospholipids, which is converted by downstream metabolic enzymes to eicosanoids [14]. cPLA2 may function as a crucial upstream regulator of the eicosanoid production for airway resistance during allergic inflammation and is responsible for the process of asthma [15]. In fact, there was a remarkable elevation of alveolar thickening observed in the cPLA2 knockout mice [16]. Recent investigations have refreshed interest in the role of prostanoids in allergic airway diseases. The COX expression is enhanced in the airways of asthmatics and they act as important regulators of normal lung architecture and function [15,17]. COX metabolites are known to influence airway tone and inflammatory responses in the lung [15]. COX2 is markedly induced in airway smooth muscle cells by pro-inflammatory cytokines and other mediators that exist in asthmatic airways [18]. The levels of prostanoids in bronchoalveolar lavage fluid are increased in asthma [19]. COX-derived PGD2 and PGF2α from arachidonic acids have been implicated in the pathogenesis of airway fibrotic process [20,21]. Accordingly, the inhibition of cPLA2- and COX2-mediated inflammatory and asthmatic pathways may provide a therapeutic clue to airway and lung injury.

Protein kinase C (PKC) enzymes are important signaling intermediates provoking airway inflammation, bronchospasm, and mucous production in asthma and chronic obstructive pulmonary disease [22]. Activated mitogen-activated protein kinases (MAPK) may be involved in modifying airway smooth muscle contraction and remodeling [23]. PKC and extracellular signal-regulated kinase (ERK) induce the phosphorylation of cPLA2 by proinflammatory cytokines in primary human bronchial epithelial cells [24]. Thus, the activity inhibition of PKC and MAPK via pharmacological or genetic approaches may block allergic inflammation of airways. The activation of PKCα reduces collagen expression via ERK signaling cascade in lung fibroblasts [25]. The ERK and p38 MAPK pathways contribute to interleukin (IL)-1β-induced COX2 expression and prostaglandin E2 (PGE2) synthesis in airway smooth muscle cells [26]. Furthermore, c-Src tyrosine kinase and MAPK regulate COX2/PGE2/IL-6-dependent airway inflammation in cigarette smoke extract-exposed tracheal smooth muscle cells [27]. To effectively and specifically block Src protein tyrosine kinase could have a great clinical implication for lung diseases as underlying mechanisms.

**Figure 1:**
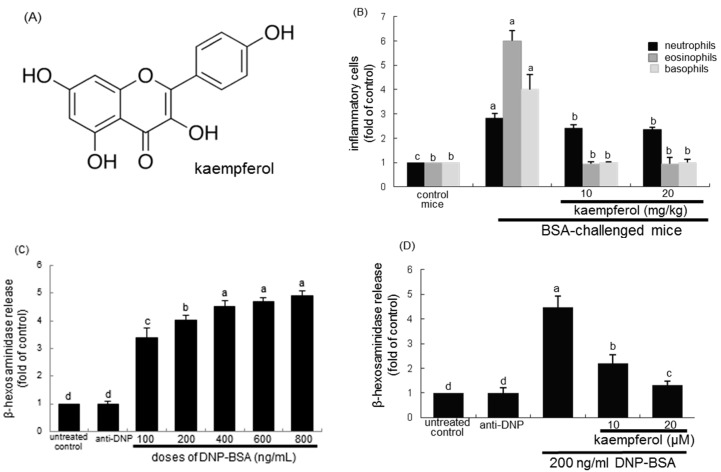
Chemical structure of kaempferol (**A**); reduction of inflammatory cells by kaempferol (**B**); and dose response of β-hexosaminidase release (**C**) and inhibitory effects of kaempferol on β-hexosaminidase release (**D**). For *in vivo* experiments, bovine serum albumin (BSA)-sensitized mice on days zero and 14 were challenged with 5% BSA aerosol on days 28, 29, and 30. The numbers of neutrophils, eosinophils, and basophils were counted in whole blood collected from sensitized BALB/c mice (*n* = 6, each group) 24 h after the final challenge with BSA or phosphate-buffered saline vehicle. For the measurements of β-hexosaminidase release, rat basophilic leukemia (RBL-2H3) cells were sensitized by adding 0.5 μg/mL anti-2,4-dinitrophenol (anti-DNP) and stimulated with 200 ng/mL DNP-bovine serum albumin (BSA) in the absence and presence of 10–20 μM kaempferol for 72 h. Respective values (mean ± SEM, *n* = 3) not sharing a letter are different at *p* < 0.05.

Kaempferol (3,4′,5,7-tetrahydroxyflavone; Figure 1A), a common flavone-type flavonoid, can be isolated from fruits and vegetables and may modulate inflammatory responses [28,29,30]. In our previous study, kaempferol inhibited airway epithelial-to-mesenchymal transition and fibrosis in endotoxin-induced epithelial cells and ovalbumin (OVA)-sensitized mice, targeting asthmatic airway constriction [31]. In addition, this compound blocked airway inflammation via disturbing tyrosine kinase/signal transducers and activators of transcription (Tyk-STAT) signaling in airway epithelial cells and in asthmatic mice [32]. The current study attempted to reveal that kaempferol dampened COX2 pathway-dependent airway thickening in 5% bovine serum albumin (BSA)-challenged BALB/c mice. This study further investigated that kaempferol inhibited acute β-hexosaminidase release and PG production in 2,4-dinitrophenyl (DNP)-BSA-challenged rat basophil leukemic RBL-2H3 cells. This study found that kaempferol may retard acute COX2-dependent allergic responses of airway wall thickening mediated by mast cells in antigen-exposed airways.

## 2. Results

### 2.1. Effects of Kaempferol on Leukocyte Distribution and β–Hexosaminidase Release

The contents of inflammatory cells of eosinophils, basophils, and neutrophils in mouse blood samples of BSA-inhaled mice were determined. As shown in Figure 1B, the inhalation of 5% BSA to mice for 20 min highly increased the contents of these inflammatory cells. It should be noted that the content of eosinophils was enhanced by approximately six-fold. In contrast, ≥10 mg/kg kaempferol markedly reduced the contents of these inflammatory cells (Figure 1B).

To determine the proper dose of DNP-BSA for the induction of allergic responses, five different doses of DNP-BSA were applied to RBL-2H3 cells for 1 h. The release of β-hexosaminidase was gradually and significantly enhanced with increasing the DNP-BSA dose up to 800 ng/mL (Figure 1C). Based on the release of β-hexosaminidase in RBL-2H3 cells, 200 ng/mL DNP-BSA was introduced to sensitize RBL-2H3 cells for the allergic responses. The antigen DNP-BSA at 200 ng/mL caused a rapid release of β-hexosaminidase from RBL-2H3 cells approximately by 4.5-fold. In contrast, 10–20 μM kaempferol dose-dependently suppressed its release in sensitized RBL-2H3 cells (Figure 1D).

### 2.2. Inhibitory Effects of Kaempferol on Prostaglandin Release and Cyclooxygenase 2 (COX2) Induction

**Figure 2:**
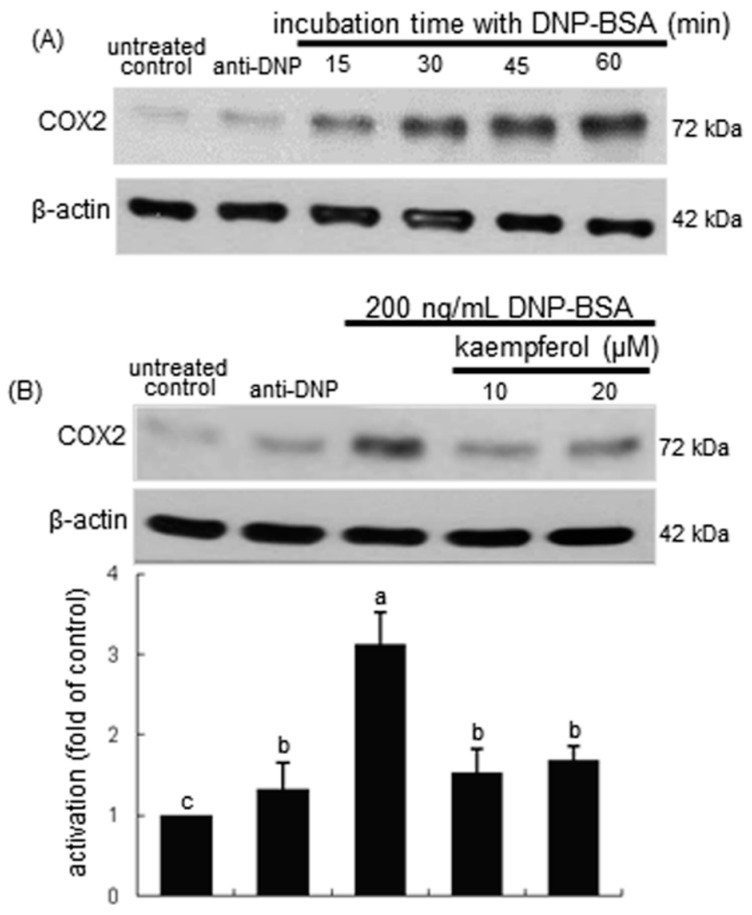
Temporal response of cyclooxygenase 2 (COX2) induction (**A**) and inhibitory effect of kaempferol on COX2 induction (**B**) in sensitized RBL-2H3 cells. Rat basophilic leukemia RBL-2H3 cells were sensitized by the addition of 0.5 μg/mL anti-2,4-dinitrophenol (anti-DNP) and stimulated for 5–60 min with 200 ng/mL DNP-bovine serum albumin (BSA) in the absence and presence of 10–20 μM kaempferol. Equal amounts of cell lysate proteins were subject to 8% SDS-PAGE and Western blot analysis with a primary antibody against COX2. The blot data (mean ± SEM, *n* = 3) in the bar graphs represent quantitative results obtained from a densitometer. Values in bar graphs not sharing a common letter refer to significantly different at *p* < 0.05.

**Figure 3:**
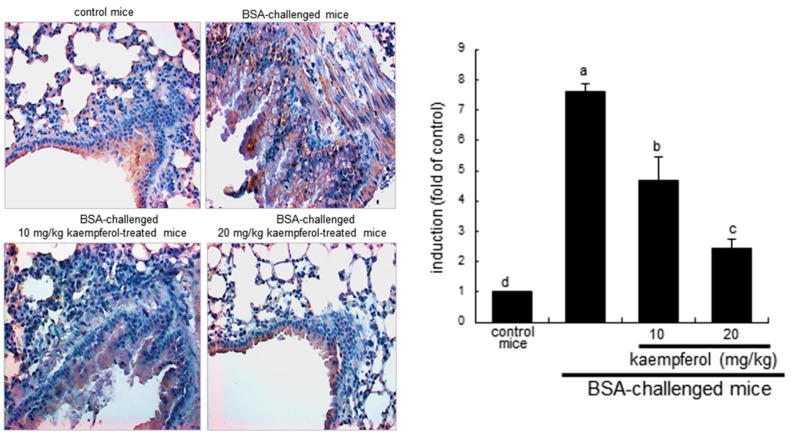
Immunohistochemical analysis showing inhibition of COX2 induction in BSA-challenged mouse airway tissues by kaempferol. BSA-sensitized mice on days zero and 14 were challenged with 5% BSA on days 28, 29, and 30. Airway tissue COX2 was visualized by staining with 3,3′-diaminobenzidine and counterstained by using hematoxylin. COX2 was identified as dark brown color and quantified by using an Axiomager optical microscope system (**right** panel). Each photograph is representative of four mice. Magnification: 200-fold. Bar graph values (mean ± SEM, *n* = 4) without a common letter differ, *p* < 0.05.

COX catalyzes the conversion of the free essential fatty acids such as arachidonic acid to prostanoids including prostaglandins and thromboxanes. This study explored that the antigen DNP-BSA induced cellular COX2 and generated prostaglandins in sensitized RBL-2H3 cells. The COX2 induction continuously increased even at 60 min-post stimulation with DNP-BSA (Figure 2A). When ≥10 μM kaempferol was added to DNP-BSA-challenged RBL-2H3 cells for 60 min, the COX2 induction was reduced (Figure 2B). The COX2 induction was confirmed in the airways of BSA-challenged BALB/c mice. There was lack of COX2 in airways of untreated control mice observed (Figure 3). The BSA inhalation to mice led to enhanced COX2 induction (dark brown staining) in mouse airway, which was reversed by oral administration of kaempferol (Figure 3). In BSA-challenged mice, there was a marked goblet cell hyperplasia and epithelial thickening observed. When 20 mg/kg kaempferol was supplemented to BSA-challenged mice, the epithelial thickening completely disappeared (Figure 3).

**Figure 4:**
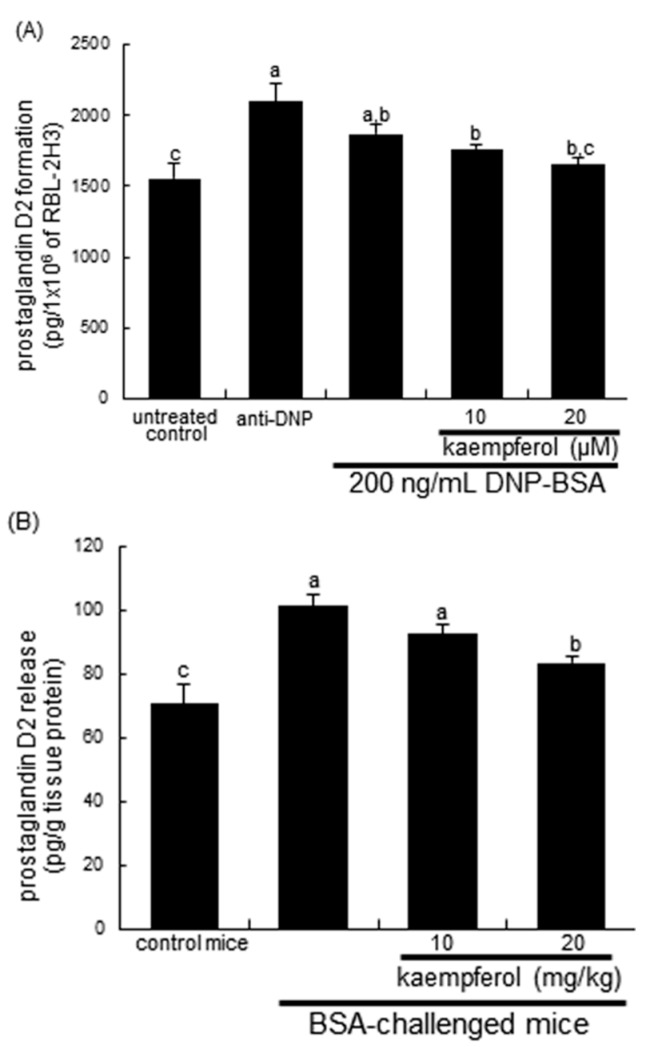

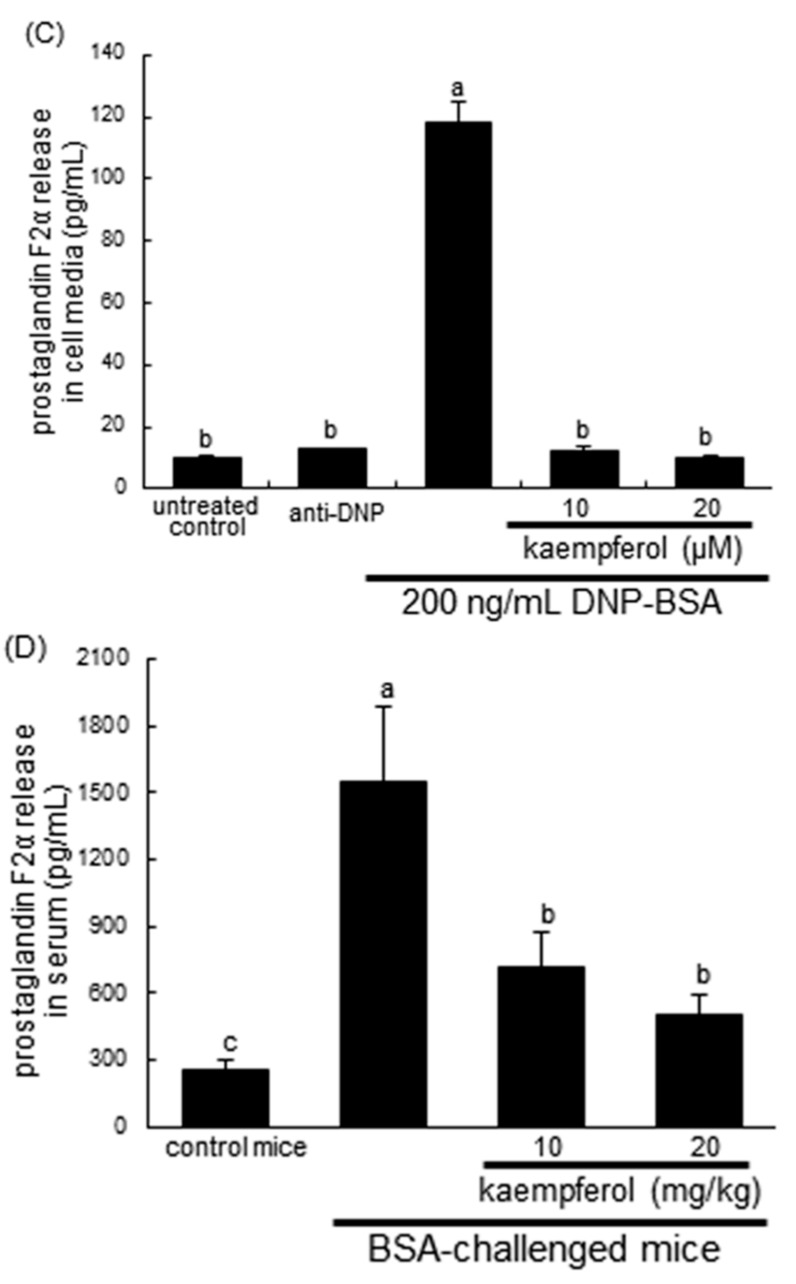
Production of prostaglandin D2 (PGD2) and prostaglandin F2α (PGF2α) in sensitized RBL-2H3 cells (**A**) and BALB/c mice (**B**). Rat basophilic leukemia RBL-2H3 cells were sensitized by the addition of 0.5 μg/mL anti-2,4-dinitrophenol (anti-DNP) and stimulated with 200 ng/mL of DNP-bovine serum albumin (BSA) in the absence and presence of 10–20 μM kaempferol. For *in vivo* experiments, BSA-sensitized mice on days 0 and 14 were challenged with 5% BSA on days 28, 29, and 30. Mast cell formation of PGD2 formation (**A**); mouse lung tissues level of PGD2 (**B**); mast cell release of PDF2α (**C**) and mouse serum level of PGF2α (**D**) were measured by using ELISA kits. Values in bar graphs not sharing a common letter refer to significantly different at *p* < 0.05.

PGD2 mainly produced by mast cells is critical to development of allergic diseases such as asthma, and PGF2α stimulates the contraction of bronchial smooth muscle [11,12,13]. In DNP IgE-sensitized cells the releases of PGD2 and PGF2α were measured in order to examine whether kaempferol may inhibit airway inflammatory response and allergic reaction. The PGD2 production was significantly enhanced in DNP-BSA-exposed RBL-2H3 cells and in BSA-inhaled mouse lung tissues (Figure 4A,B). Interestingly, the anti-DNP sensitization *per se* considerably induced the PGD2 formation in RBL-2H3 cells. In contrast, the enhanced production of PGD2 was attenuated in 20 μM kaempferol-treated DNP-BSA-sensitized cells and in 20 mg/kg kaempferol-supplemented BSA-challenged mice (Figure 4A,B). Consistently, the PGF2α release was remarkably enhanced in sensitized mast cells, which was dampened by ≥10 μM kaempferol (Figure 4C). In addition, ≥10 mg/kg kaempferol reversed the elevated serum level of PGF2α in BSA-inhaled mice (Figure 4D).

### 2.3. Inhibition of Airway Thickening by Kaempferol

**Figure 5:**
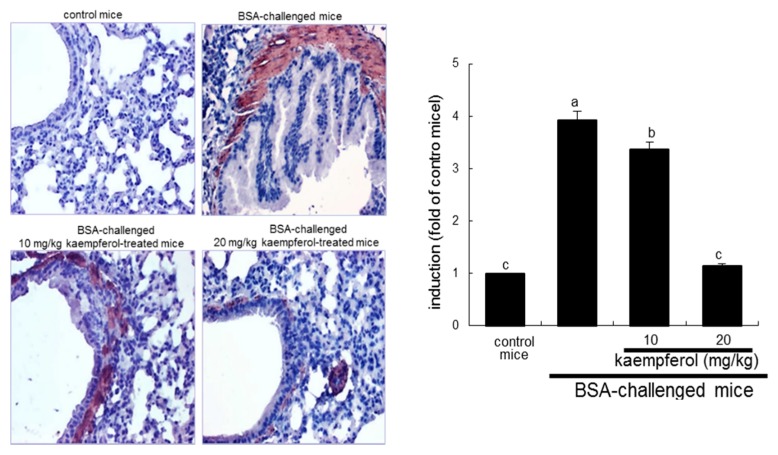
Immunohistochemical analysis showing inhibition of α-smooth muscle actin (α-SMA) induction in BSA-challenged mouse airway tissues by kaempferol. BSA-sensitized mice on days 0 and 14 were challenged with 5% BSA on days 28, 29 and 30. Airway tissue α-SMA was visualized by staining with 3,3′-diaminobenzidine and counterstained by using hematoxylin. α-SMA was identified as dark brown color and quantified by using an Axiomager optical microscope system (**right** panel). Each photograph is representative of four mice. Magnification: 200-fold. Bar graph values (mean ± SEM, *n* = 4) without a common letter differ, *p* < 0.05.

Severe asthma is characterized by airway hypertrophy and hyperplasia of smooth muscle leading to thickening of the airway wall [33]. This study investigated that kaempferol inhibited airway smooth muscle hypertrophy and hyperplasia. There was dense brown staining in mouse airway walls of BSA-challenged mice observed, evidenced by staining with anti-α-smooth muscle actin (α-SMA) and 3.3′-diaminobenzidine (Figure 5). However, the airway hypertrophy and hyperplasia were retarded by orally administrating ≥10 mg/kg kaempferol to 5% BSA-exposed mice, indicating that kaempferol may alleviate antigen-triggered bronchial thickening and constriction due to smooth muscle contraction (Figure 5).

### 2.4. Inhibition of Cytosolic Phospholipase A2 (cPLA2) Activation by Kaempferol

**Figure 6:**
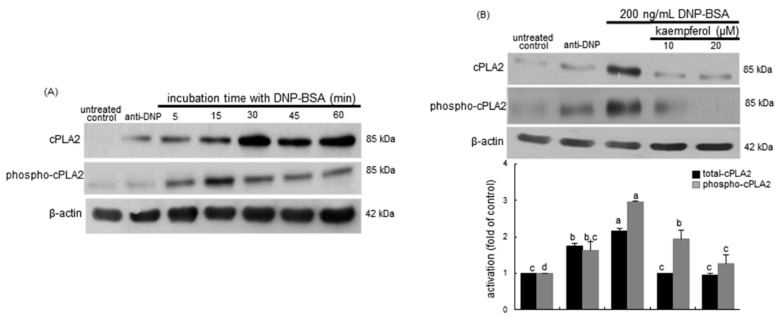
Time course response of activation of cPLA2 (**A**) and inhibitory effects of kaempferol on cellular level of phospho-cPLA2 (**B**) in sensitized RBL-2H3 cells. Rat basophilic leukemia RBL-2H3 cells were sensitized by adding 0.5 μg/mL anti-2,4-dinitrophenol (anti-DNP) and stimulated for 5–60 min with 200 ng/mL of DNP-bovine serum albumin (BSA) in the absence and presence of 10–20 μM kaempferol. Equal amounts of cell lysate proteins were subject to 8% SDS-PAGE and Western blot analysis with a primary antibody against total cPLA2 and phospho-cPLA2. β-Actin protein was used as an internal control for cellular total cPLA2 and phospho-cPLA2. The bar graphs (mean ± SEM, *n* = 3) in the bottom panel represent quantitative results obtained from a densitometer. Values not sharing a common letter refer to significantly different at *p* < 0.05.

The current study attempted to examine whether kaempferol inhibited the cPLA2 activation releasing arachidonic acid for the prostanoid production by the enzyme COX2. Figure 6A shows the time course response of cPLA2 activation to the DNP-BSA antigen in sensitized mast cells. Total cPLA2 appeared to be induced within 30 min by the stimulation with 200 ng/mL DNP-BSA. The cPLA2 phosphorylation was substantially elevated even in anti-DNP-alone-sensitized cells relative to non-sensitized controls. However, the ≥10 μM kaempferol supplementation suppressed the cPLA2 induction and phosphorylation enhanced by an exposure to DNP-BSA for 15–30 min in sensitized cells (Figure 6B). Accordingly, kaempferol may inhibit the release of arachidonic acid from membrane phospholipids under allergic circumstances.

### 2.5. Blockade of Syk-ERK (Extracellular Signal-Regulated Kinase) Signaling by Kaempferol

**Figure 7:**
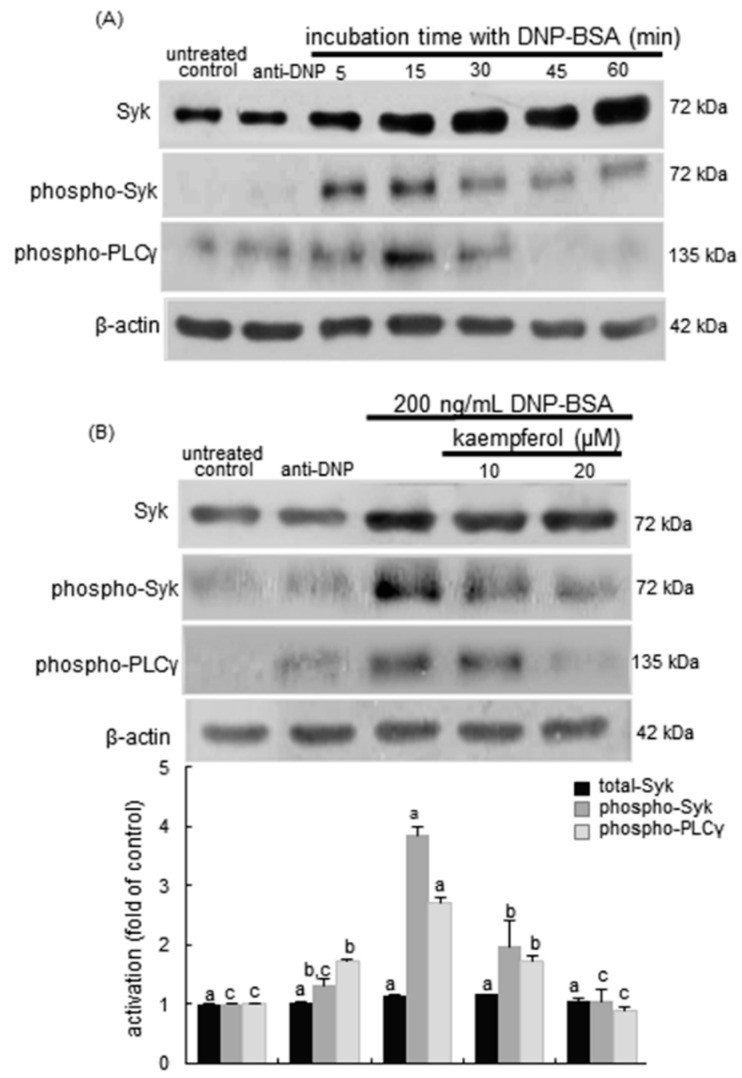
Temporal response of the activation of Syk and phospholipase Cγ (PLCγ) (**A**) and inhibitory effects of kaempferol on the activation of Syk and PLCγ (**B**) in RBL-2H3 cells. Rat basophilic leukemia RBL-2H3 cells were sensitized by adding 0.5 μg/mL anti-2,4-dinitrophenol (anti-DNP) and stimulated for 5–60 min with 200 ng/mL of DNP-bovine serum albumin (BSA) in the absence and presence of 10–20 μM kaempferol. Equal amounts of cell lysate proteins were subject to 8% SDS-PAGE and Western blot analysis with a primary antibody against total Syk, phospho-Syk and phospho-PLCγ (three separate experiments). β-Actin protein was used as an internal control for cellular total Syk, phospho-Syk, and phospho-PLCγ. The bar graphs (mean ± SEM) in the bottom panel represent quantitative results obtained from a densitometer. Values not sharing a common letter refer to significantly different at *p* < 0.05.

Syk is a vital regulatory factor in the IgE-mediated allergic signal pathway in mast cells and basophils [34]. Syk is phosphorylated on many tyrosines following the binding of IgE-allergen complexes to Fc receptors leading to initiation of inflammatory signaling via downstream proteins. This study investigated that kaempferol blocked the Syk-PLCγ signaling responsible for the allergic responses in DNP-BSA-sensitized RBL-2H3 cells. There was lack of Syk activation in anti-DNP-alone-sensitized mast cells (Figure 7A). The DNP-BSA challenge prompted the activation of Syk and PLCγ within 5–15 min and such activation was reduced after 30 min post-stimulation (Figure 7A). When 10–20 μM kaempferol was supplemented to DNP-BSA-challenged RBL-2H3 cells for 15 min, the activation of Syk and PLCγ was highly attenuated (Figure 7B). It should be noted that total Syk was not significantly influenced by the antigen or kaempferol in sensitized mast cells.

**Figure 8:**
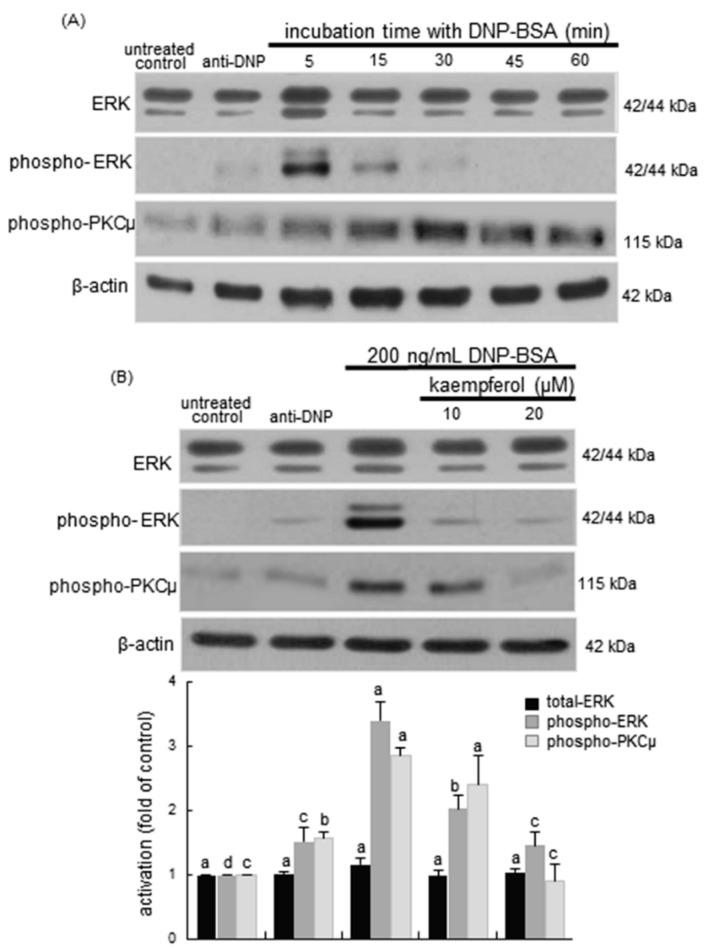
Time course responses of the activation of protein kinase Cμ (PKCμ) and extracellular signal-regulated kinase (ERK) (**A**); and inhibitory effects of kaempferol on cellular levels of total ERK, phospho-ERK and phospho-PKCμ (**B**) in sensitized RBL-2H3 cells. Rat basophilic leukemia RBL-2H3 cells were sensitized by addition of 0.5 μg/mL anti-2,4-dinitrophenol (anti-DNP) and stimulated for 5–60 min with 200 ng/mL of DNP-bovine serum albumin (BSA) in the absence and presence of 10–20 μM kaempferol. Equal amounts of cell lysate proteins were subject to 8% SDS-PAGE and Western blot analysis with a primary antibody against total ERK, phospho-ERK, and phospho-PKCμ. β-Actin protein was used as an internal control for total ERK, phospho-ERK, and phospho-PKCμ. The bar graphs (mean ± SEM, *n* = 3) in the bottom panel represent quantitative results obtained from a densitometer. Values not sharing a common letter refer to significantly different at *p* < 0.05.

This study further investigated the PKCμ-ERK downstream signaling for the cPLA2 activation in kaempferol-supplemented RBL-2H3 cells exposed to the antigen DNP-BSA. The phosphorylation of ERK was rapidly enhanced by the antigen within 5–15 min and such activation was diminished after 30 min post-stimulation (Figure 8A). The PKCμ activation was elevated in antigen-treated sensitized cells in 15–30 min. In contrast, the ERK activation by the antigen was completely dampened by treating submicromolar kaempferol to sensitized RBL-2H3 cells for 5 min (Figure 8B). Additionally, the PKCμ activation was attenuated by supplementing 10–20 μM kaempferol to sensitized RBL-2H3 cells challenged with DNP-BSA for 30 min (Figure 8B). Collectively, these results suggest that kaempferol may block the Syk-PLCγ-PKCμ-ERK axis signaling for the cPLA2 activation.

## 3. Discussion

Nine major findings were extracted from this study. (1) Oral administration of kaempferol markedly reduced the number of eosinophils and basophils in blood; (2) adding kaempferol diminished the β-hexosaminidase release enhanced in DNP-BSA-sensitized RBL-2H3 cells; (3) kaempferol reduced the COX2 induction upregulated in DNP-BSA-challenged RBL-2H3 cells and in the airways of BSA-challenged BALB/c mice; (4) goblet cell hyperplasia and airway thickening by the antigen BSA were alleviated in kaempferol-treated mice; (5) kaempferol reversed the production of PGD2 and PGF2α elevated in DNP-BSA-exposed mast cells and reduced the lung tissue and serum levels of these PGs in BSA-challenged mice; (6) the BSA challenge prompted the α-SMA induction in mouse airway smooth muscle, which was attenuated by orally administrating kaempferol to mice; (7) the kaempferol supplementation inhibited the cPLA2 activation in DNP-BSA-sensitized cells, indicating that this compound may inhibit the release of arachidonic acid from membrane phospholipids; (8) the activation of Syk-PLCγ pathway was suppressed in kaempferol-supplemented DNP-BSA-challenged mast cells; and (9) the rapid activation of PKCμ and ERK by the antigen was dampened by adding submicromolar kaempferol to BSA-sensitized mast cells. Accordingly, kaempferol may retard acute allergic responses leading to airway wall thickening through interfering with the COX2-mediated pathway in antigen-exposed mast cells.

Kaempferol has been shown to antagonize allergic reactions in a murine allergic model [35,36]. Our previous studies found that kaempferol ameliorated allergic inflammation in airway epithelial cells and in mice with allergic asthma through disturbing the signaling pathways of nuclear factor (NF)-κB and Tyk-STAT [32,36]. This compound inhibited allergic responses via the regulation of the production of IL-32 and thymic stromal lymphopoietin and nasal mucosa caspase-1 activity in a murine allergic rhinitis model [35]. Other investigation has shown that kaempferol blocks the secretion of allergic mediators in RBL-2H3 cells and suppresses IgE-OVA-induced ERK activation and chemokine release [37]. Similarly, kaempferol abrogated the increase in Th2 cytokines and tumor necrosis factor-α levels through inhibiting the Akt activation in a mouse model of allergic asthma [38]. Consistently, this study revealed that kaempferol inhibited acute release of β-hexosaminidase in DNP-BSA-exposed basophilic mast cells and reduced increased number of inflammatory cells in BSA-challenged sensitized mice. Thus, kaempferol may be a potent anti-allergic compound targeting allergic asthma typical of airway inflammation.

Airway smooth muscle is the key ultimate effector of acute airway narrowing due to contraction, as it comprises a significant proportion of the airway wall. Airway smooth muscle contraction, in combination with mechanical transduction and contraction-inflammation synergies, contributes to the process of asthma [39]. Thus, the inhibition of bronchial constriction is a prominent therapeutic strategy antagonizing asthma pathogenesis. In the current study the BSA inhalation caused airway goblet cell hyperplasia and increased the thickness of airway smooth muscle layers in mice. Kaempferol suppressed the hyperplasia and hypertrophy of epithelial cells and smooth muscle cells in airways, indicating that kaempferol may block the airway narrowing and smooth muscle contraction. This study postulated that the hypertrophy of airway smooth muscle in BSA-sensitized mice occurred via a COX2-dependent mechanism mediated by mast cells in airways. The mast cell infiltration of the airway smooth muscle increases during asthmatic exacerbation following allergen inhalation [40,41]. Airway smooth muscle promotes mast cell chemotaxis through the secretion of a wide array of chemotactic factors [42]. Moreover, mast cells play an important role in airway responses to allergens through the release of histamine, a potent mediator of bronchoconstriction. The inhibition of the histamine release by kaempferol from mast cells may be responsible for promoting airway smooth muscle hyperplasia and contraction in BSA-sensitized mice.

Activation of mast cells produces prostanoids including PGs and thromboxanes from arachidonic acid by the action of COX2 in allergic disorders, which causes airway smooth muscle contraction [10,11]. It is assumed that mediators degranulated from mast cells such as histamine and tryptase induce significant increases in COX2 expression and prostanoid formation, which may contribute to the increase in muscle mass evident in asthmatic airways [43]. The COX metabolites are known to influence airway tone and inflammatory responses in the lung [15]. This study found that kaempferol dampened the COX2 induction in DNP-BSA-sensitized mast cells and in lung tissues of BSA-inhaled mice. In addition, this compound reduced the production of PGD2 and PGF2α from sensitized mast cells and encumbered the PG production in sensitized mice. It can be deemed that the inhibition of airway narrowing and smooth muscle cell proliferation by kaempferol was attributed to the blockade of the prostanoid release in airways by mast cells and resident smooth muscle cells. The COX2-dependent production of PGD2 and PGF2α are known to be crucial to the development of asthmatic responses featuring the mucus secretion in bronchi and trachea and the bronchial smooth muscle contraction [12,13].

cPLA2 functions as a critical upstream regulator of the eicosanoid production and has been implicated in asthmatic responses to airway resistance [15]. The remarkable elevation of bronchial and alveolar thickening is observed in cPLA2 knockout mice [16]. Thus, the blockade of cPLA2 mediation may provide a therapeutic approach to airway and lung injury. One investigation has shown that the cPLA2α inhibition can attenuate PGD2 release and adenosine monophosphate-induced contraction of human bronchi in anti-IgE-stimulated human lung mast cells and late-phase bronchoconstriction in a sheep model of allergic inflammation [44]. These findings suggest that the inhibition of cPLA2 with kaempferol may represent a new therapeutic option for the treatment of asthma. On the other hand, kaempferol interrupted the activation of the PKCμ, ERK and cPLA2 axis pathway for the COX2 mediation in DNP-BSA-sensitized mast cells. In fact, PKC and ERK induce the phosphorylation of cPLA2 by pro-inflammatory cytokines leading to the modification of airway smooth muscle contraction and remodeling [23,24]. Accordingly, the inhibition of PKCμ and ERK by dietary kaempferol may block the narrowing and thickening of airways. In addition, this study showed that kaempferol deterred the Syk signaling responsible for PLCγ activation. Similarly, the natural compound homoisoflavanone isolated from *Cremastra appendiculata* Makino down-regulates the PGD2 production in IgE/antigen-stimulated mast cells through blocking Syk signaling together with the inhibition of cPLA2 [45]. It has been shown that the Syk inhibition blocks the mast cell degranulation in airways and the early- and late-phase asthmatic responses. Moreover, Syk is a key activator of signaling downstream of multiple surface receptors implicated in asthma, supporting a key role for Syk signaling in mediating allergic airway responses [46,47] Thus, kaempferol may abrogate COX2-mediated airway thickening by hampering Syk-PLCγ-cPLA2 signaling of antigen-exposed mast cells.

## 4. Materials and Methods

### 4.1. Materials

Eagle’s Minimum Essential Medium (EMEM) and other cell culture supplies, including fetal bovine serum (FBS), penicillin-streptomycin and trypsin-EDTA were obtained from ThermoFisher Scientific (Waltham, MA, USA) or Lonza (Walkersville, MD, USA). Both mouse monoclonal DNP antibody and DNP-BSA were provided by Sigma-Aldrich Chemicals (St. Louis, MO, USA). All antibodies against total Syk, phospho-Syk, phospho-PKCμ, phospho-PLCγ, total ERK, phospho-ERK, total cPLA2, phospho-cPLA2 and COX2 were purchased from either Cell Signaling Technology (Boston, MA, USA) or Santa Cruz Biotechnology (Santa Cruz, CA, USA). Horseradish peroxidase (HRP)-conjugated goat anti-mouse IgG and goat anti-rabbit IgG were purchased from Jackson ImmunoResearch Laboratories (West Grove, PA, USA).

### 4.2. Cell Culture and Kaempferol Treatment

Rat basophilic leukemia (RBL-2H3) cells were acquired from the American Type Culture Collection (ATCC, Manassas, VA, USA). RBL-2H3 cells were cultured in EMEM containing 10% FBS, 100 μg/mL streptomycin, 2 mM l-glutamine and 100 U/mL penicillin and then incubated at 37 °C with 5% CO_2_ in air. RBL-2H3 cells sensitized by adding 0.5 μg/mL anti-DNP to cells in order to generate IgE-mediated allergic responses. The sensitized RBL-2H3 cells were then washed twice using a siraganian buffer containing 25 mM piperazine-*N*,*N′*-bis(2-ethanesulfonic acid), 0.4 mM MgCl_2_, 40 mM NaOH, 119 mM NaCl, 1 mM CaCl_2_, 5 mM KCl, 5.5 mM glucose, and 1% BSA (pH 7.4). The RBL-2H3-sensitized cells were treated with 10–20 μM kaempferol for 1 h, followed by the addition of 200 ng/mL DNP-BSA for 5–60 min. All media and cells were then collected and stored at −70 °C until use.

### 4.3. Murine Animal Model

Three-week-old male BALB/c mice were randomly assigned to the four treatment groups as follows (*n* = 8 per group). (1) PBS-sensitized mice; (2) BSA-sensitized mice; (3) BSA-sensitized and 10 mg/kg kaempferol-administered mice; and (4) BSA-sensitized and 20 mg/kg kaempferol-administered mice. Mice were given a commercial mouse chow diet containing 20.5% protein, 3.5% fat, 8% fiber, 8% ash, and 0.5% phosphorus and were allowed access to food and water *ad libitum*. The mice were kept under a 12 h light and dark cycle at 23 ± 1 °C with 50% ± 5% relative humidity in specific pathogen-free conditions at the Animal Facility of Hallym University. Mice were allowed to become accustomed to their surroundings for one week before starting the allergic experiments. Sensitization of all experimental mice was carried out by subcutaneous injection with 20 μg BSA in 30 μL PBS and 50 μL Imject Alum on days 0 and 14. The control mice were injected with a combination of 50 μL PBS and 50 μL Imject Alum without BSA. On days 28, 29, and 30, only the experimental mice sensitized to BSA were subject to inhalation of 5% BSA, while control mice were challenged with 5% PBS for 20 min in a plastic chamber connected to a Medel aerosol nebulizer (Parma, Italy). All mice were sacrificed 24 h after the last challenge. Whole blood samples were directly used to measure the contents of eosinophils, basophils and neutrophils (Hemavet HV950 Multispecies Hematologic Analyzer; Drew Scientific, Dallas, TX, USA). The right lung was stored in 4% paraformaldehyde until use.

All experiments were approved by the Committee on Animal Experimentation of Hallym University and performed in compliance with the University’s Guidelines for the Care and Use of Laboratory Animals (Hallym 2014-37; 9 June 2014). No mice died during experiments, and no obvious warnings of mouse exhaustion were observed.

### 4.4. β-Hexosaminidase Analysis

Mediators are released as a consequence of immediate degranulation, upon mast cell activation. To monitor mast cell degranulation, the release of the granule component, β-hexosaminidase was measured in sensitized RBL-2H3 cells. All culture media were collected and then stored until analysis. Briefly, 20 μL of each culture medium was mixed with 1 mM *p*-nitrophenyl-*N*-acetyl-β-d-glucosaminide in 0.1 M citrate buffer (pH 4.5) and then incubated at 37 °C for 1 h. After incubation, 200 μL of 0.1 M Na_2_CO_3_ and NaHCO_3_ mixture was applied to stop the reactions. The absorbance of each sample was then measured at λ = 405 nm.

### 4.5. Western Blot Analysis

Whole RBL-2H3 cells were lysed with 1 M Tris-HCl (pH 6.8) lysis buffer containing 10% SDS, 1% glycerophosphate, 0.1 M Na_3_VO_4_, 0.5 M NaF and protease inhibitor cocktail. Equivalent amounts of proteins from each cell lysate were then electrophoresed on 6%–15% SDS-PAGE and transferred onto nitrocellulose membranes. After blocking for 3 h with 5% nonfat dry milk to avoid nonspecific binding, each membrane was incubated overnight at 4 °C with a specific primary antibody against total Syk, phospho-Syk, phospho-PLCγ, phospho-PKCμ, total ERK, phospho-ERK, total cPLA2, phospho-cPLA2, or COX2. The HRP-conjugated secondary antibody was then incubated for 1 h, after which Supersignal West Pico Chemiluminescent substrate (Waltham, MA, USA) and Agfa medical X-ray film blue (Mortsel, Belgium) were used for the protein detection.

### 4.6. Immunohistochemical Analysis

All right lungs fixed in 4% paraformaldehyde were paraffin-embedded and then sectioned into 5 μm thickness. Each lung tissue section was placed onto a microscope slide and allowed to dry overnight at room temperature. The deparaffinization and rehydration of each lung section were then completed using xylene and alcohol. All deparaffinized and rehydrated sections were boiled in sodium citrate buffer (10 mM sodium citrate, 0.05% Tween20, pH 6.0) for antigen retrieval. Application of COX2 antibody or α-SMA antibody was performed overnight at 4 °C, and then the incubation with HRP-conjugated anti-goat or anti-mouse IgG was conducted for 1 h. Consecutively, the lung tissues were developed by staining with 3.3′-diaminobenzidine (brown color), followed by the counter-staining using hematoxylin. Each slide was then mounted in VectaMount mounting medium (Vector Laboratories, Burlingame, CA, USA), and final images of each lung tissue sections were taken by an optical microscope system (Axiomager, Zeiss, Oberkochen, Germany).

### 4.7. Enzyme-Linked Immunosorbent Assay (ELISA)

The release of PGD2 and PGF2α in sensitized RBL-2H3 cells, and the lung tissue PGD2 level and the serum PGF2α level in BALB/c mice, were examined by using ELISA kits of PGD2 (Cayman Chemical, Ann Arbor, MI, USA) and PGF2α (Abcam, Cambridge, UK), according to the manufacturer’s instructions.

### 4.8. Statistical Analysis

The data are presented as mean ± SEM for each treatment group in the *in vivo* and *in vitro* experiments. Statistical analyses were conducted using a Statistical Analysis Systems program (SAS Institute, Cary, NC, USA). One-way ANOVA was used to determine inhibitory the effects of kaempferol on allergic responses in mast cells and sensitized mice. Differences among treatment groups were analyzed with Duncan’s multiple-range test and were considered to be significant at *p* < 0.05.

**Figure 9:**
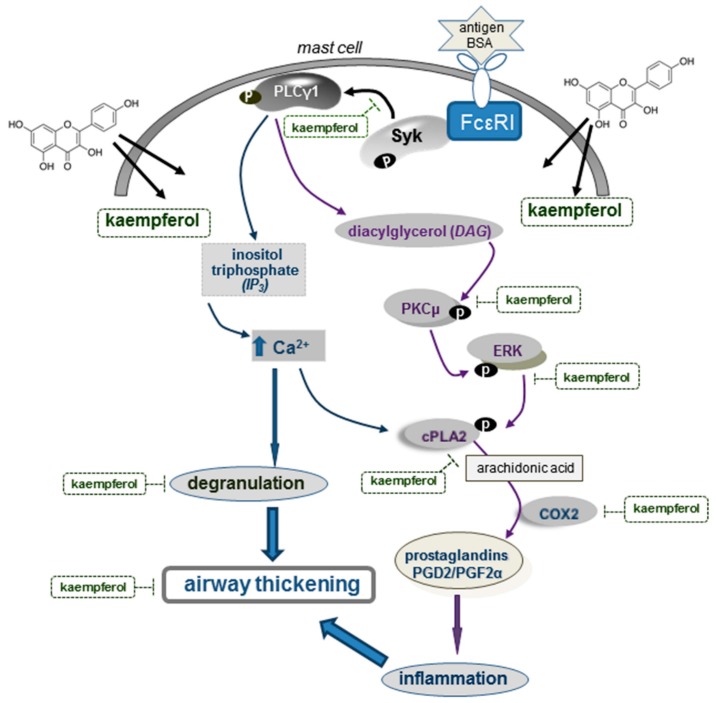
Schematic diagram showing the inhibitory effects of kaempferol on allergic degranulation and inflammation and its mechanistic actions in antigen-induced airway thickening. As depicted, kaempferol inhibits the direct allergic signaling cascades induced by the antigen BSA. P denotes phosphorylation. All the arrows indicate increase, activation or induction; ┥ indicates inhibition or blockade.

## 5. Conclusions

This study investigated the potential of kaempferol as a target for therapeutic strategies in preventing asthmatic diseases. Non-toxic kaempferol-suppressed mast cell degranulation and COX2-mediated production of PGD2 and PGF2α in DNP-BSA-challenged sensitized mast cells. Oral administration of kaempferol blocked the BSA inhalation-induced epithelial cell excrescence and airway thickening by suppressing the induction of COX2 and α-SMA in mouse airways. Moreover, kaempferol inhibited the lung tissue and serum levels of PGD2 and PGF2α in mice exposed to BSA. Furthermore, the kaempferol supplementation deterred the mast cell activation of cPLA2 responsive to PKCμ and ERK activated by DNP-BSA. The activation of Syk-PLCγ pathway was suppressed in kaempferol-supplemented DNP-BSA-challenged mast cells. Therefore, kaemepferol was effective in ameliorating epithelial thickening and airway smooth muscle hypertrophy through disturbing Syk-PLCγ signaling and PKCμ-cPLA2-COX2 signaling in antigen-challenged mast cells (Figure 9).
